# A Peptide-Based Enzyme-Linked Immunosorbent Assay for Detecting Antibodies Against Avian Infectious Bronchitis Virus

**DOI:** 10.3389/fvets.2020.619601

**Published:** 2021-01-21

**Authors:** Liping Yin, Qi Wu, Zhixian Lin, Kun Qian, Hongxia Shao, Zhimin Wan, Yuelong Liu, Jianqiang Ye, Aijian Qin

**Affiliations:** ^1^Key Laboratory of Avian Preventive Medicine, Ministry of Education, Yangzhou University, Yangzhou, China; ^2^JiangsuLihua Animal Husbandry Co., Ltd, Changzhou, China; ^3^Jiangsu Co-innovation Center for Prevention and Control of Important Animal Infectious Diseases and Zoonoses, Yangzhou, China; ^4^The International Joint Laboratory for Cooperation in Agriculture and Agricultural Product Safety, Ministry of Education, Yangzhou University, Yangzhou, China

**Keywords:** infectious bronchitis virus, pELISA, antibody, detection, chicken

## Abstract

Infectious bronchitis virus (IBV) causes substantial loss to the poultry industry despite extensive vaccination. Assessing the antibody response is important for the development and evaluation of effective vaccines. We have developed an enzyme-linked immunosorbent assay (ELISA) for the detection of IBV-specific antibodies, using a synthetic peptide based on a conserved sequence in the IBV spike protein. This peptide-based ELISA (pELISA) specifically detects antibodies to different genotypes of IBV but not antibodies against other common chicken viruses. This assay could detect IBV-specific antibody response on as early as day 7 postinfection. In the testing with field serum samples collected from chickens administered with IBV vaccines, the sensitivity, specificity, and accuracy of pELISA were 98.30, 94.12, and 98.8%, respectively, relative to indirect immunofluorescence assay. Our data demonstrate that the pELISA is of value for the detection of IBV antibody and the evaluation of IBV vaccines.

## Introduction

Avian infectious bronchitis, a highly contagious disease, is caused by a coronavirus, that is, infectious bronchitis virus (IBV). The infection of IBV generally causes serious respiratory and renal diseases in broilers and lowers egg production in layers (1), resulting in significant economic loss in the poultry industry (2). Although the efficacy is far from optimal, vaccines represent one of the most effective tools for the control of IBV. As for other animal and human vaccines, assessment of antibody response is of key importance for IBV vaccine development.

The IBV genome encodes four structural proteins as well as at least 15 non-structural and accessory proteins (1). Among these proteins, the surface spike (S) glycoprotein is the major antigen that induces protective immune response against IBV (3). The S protein consists of two subunits, S1 and S2, with the S1 subunit being responsible for binding cellular receptors (4) and the major target of neutralizing antibodies. The S2 subunit is more conserved than S1 and also plays a role in inducing protective immune response (5–7), as well as facilitating membrane fusion and viral entry (5, 8, 9). It has been reported that S2 could produce cross-protection against strains that differ in their S1 subunits (7).

A feasible and practical immunoassay for antibody detection and immune response measurement is critical for vaccine development. Enzyme-linked immunosorbent assays (ELISAs) based on whole IBV viral particles, as well as recombinant S1, nucleocapsid, and non-structural proteins, have been reported for detecting antibodies against IBV (10–13). Although these assays have achieved promising results, they have some limitations, especially in detecting antibodies induced by emergent or variant IBV strains.

Our previous studies revealed an epitope in S2 and identified the key amino acids in this epitope (14). Based on this finding, we have designed an IBV S2-based peptide and developed an ELISA for the detection of antibodies against IBV.

## Materials and Methods

### Synthetic Peptide and Serum Samples

A 20-mer peptide, SCPYVSYGRFCIQPDGSIKQ, corresponding to amino acid positions 8 to 27 on the S2 protein of IBV CK/CH/2010/JT1 strain (GenBank KU361187), was synthesized (Synpeptide Co., Ltd., Shanghai, China) and used as the coating antigen for the peptide-based ELISA (pELISA). Serum samples that were used in our study included 100 serum samples collected from specific-pathogen-free (SPF) chickens (Spirax Ferrer Poultry Science and Technology Co., Ltd., Jinan, China), 250 serum samples collected from chickens that were vaccinated with IBV vaccines H120 and H52 (Lihua Animal Husbandry Co., LTD, Jiangsu, China), and sera against IBV strains Massachusetts 41 (M41), 4/91, H52, H120, and CK/CH/2010/JT1, which were prepared in our laboratory by infecting SPF chickens with 1,000 median egg infectious dose (EID_50_) of each strain. Immune serum against QXL87 (GenBank accession no. MH743141) vaccine strain (QX-type) was obtained from Zhongchong Sino Biological Technology Co., Ltd. (Shanghai China). The other sera were kept in our laboratory, which were made from SPF chickens infected with the viruses (15).

### pELISA Procedure

For the pELISA, 96-well polystyrene plates were coated with 0.63 μg/ml of the synthetic peptide in 0.1 M carbonate buffer (pH 9.6) at 4°C overnight. After washing with phosphate-buffered saline containing 0.05% vol/vol Tween 20 (PBST), the plates were blocked with 300 μl/well of 8% rabbit serum in PBST (Lanzhou Minhai Biological Engineering Co., Ltd., China) for 3 h at 37°C. Following three washes with PBST, 100 μl serum (1:200 dilution in PBST) was added to the wells, and the plates were incubated at 37°C for 1 h. The plates were then washed five times with PBST and further incubated with 100 μl/well horseradish peroxidase–conjugated goat anti–chicken immunoglobulin G (IgG) (1:20,000) (Jackson ImmunoResearch Laboratories, Inc., USA) for 1 h at 37°C. After washing for five times, the signals were developed by incubating the plates with 100 μL/well TMB substrate for 20 min at 37°C, followed by stopping the color development with 100 μl/well of 1% sodium dodecyl sulfate. OD_650_ values were read with an ELISA reader (BioTek, VT, USA). Each assay was repeated twice.

For the commercial IBV antibody ELISA kit (IDEXX, Westbrook, ME), the assay was performed according to the manufacturer's protocol. Briefly, 100 μl of 1:500 diluted sera was added to each well. After incubation for 30 min at room temperature and washing for three times, 100 μl conjugated antibody was added and incubated for 30 min at room temperature. The plates were washed five times, and the substrate was added in the well for color development for 15 min and terminated with stop solution. OD_650_ values were read with an ELISA reader (BioTek, VT, USA).

### Cutoff Value, Specificity, and Reproducibility

After the optimal dilution and peptide concentration were set, 100 negative serum samples from SPF chickens were tested by pELISA to set a cutoff value. To examine the specificity of the pELISA, positive serum samples against avian influenza virus (AIV), avian leukemia virus (ALV), Newcastle disease virus (NDV), infectious bursal disease virus (IBDV), Marek's disease virus (MDV), and egg drop syndrome virus (EDS-76V) were tested. Evaluation of the assay reproducibility within and between runs was performed with eight serum samples, four positive and another four negative, which were confirmed in indirect immunofluorescence assay (IFA). For intra-assay (within-plate) reproducibility, four replicates of each serum sample were analyzed within the same plate, and the experiment was performed three times independently. For interassay (between-run) reproducibility, four replicates of each serum sample were run in different plates. This test was performed three times using plates coated at different times. The mean OD_650_ value, standard deviation (SD), and coefficient of variation (CV) were calculated.

### Performance of the pELISA

To further evaluate the pELISA, 250 serum samples from vaccinated chickens were tested by pELISA, IFA, and the commercial ELISA kit (IDEXX, USA). The IFA was conducted according to the previously reported protocol (16). Briefly, primary chicken embryo kidney cells were grown in the 96-well plates and infected with IBV M41 strain. After 2 days, the cells were fixed with acetone and alcohol (3:2) for 5 min. The sera diluted 1:200 with PBS were added to the wells and incubated for 60 min. After washing with PBST, cells were incubated with the fluorescein isothiocyanate–conjugated rabbit–anti-chicken IgG for 60 min, followed by washing five times and observation under the fluorescence microscope. The sensitivity, specificity, and the accuracy of the pELISA and commercial ELISA kit were evaluated by comparing to the data generated by IFA.

To further evaluate the assay, five 6-week-old SPF chickens were vaccinated intranasally with 10^3^ EID_50_ IBV strain H52 or 4/91; sera were collected on days 3, 7, 14, 21, and 28 and tested by the pELISA and the commercial IDEXX ELISA kit.

## Results

### Optimization of the pELISA

**Figure 1:**
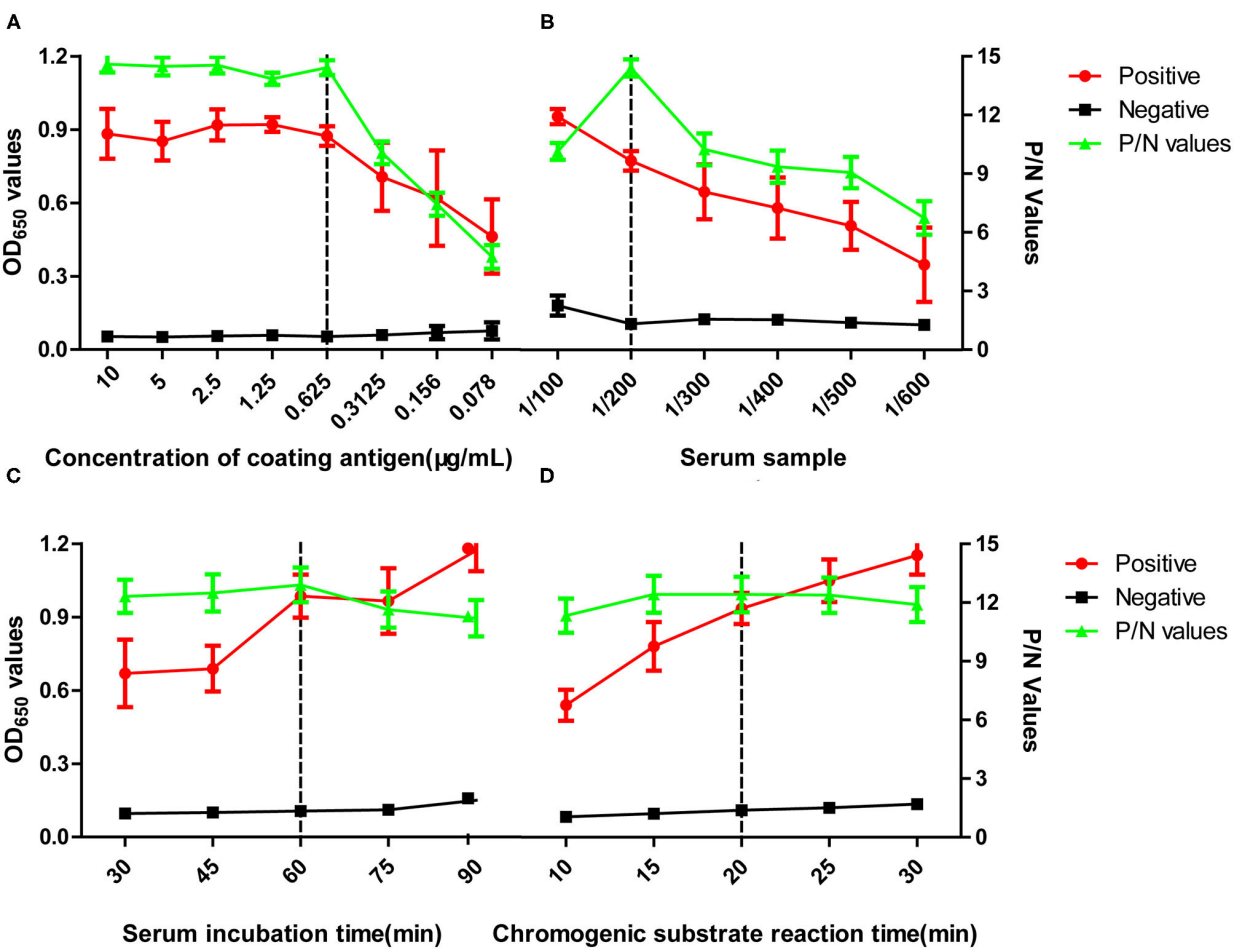
Optimization of pELISA. Assays were performed to optimize **(A)** concentration of the coating antigen, **(B)** dilution of the serum sample, **(C)** incubation time for the serum sample, **(D)** incubation time for the TMB substrate. The vertical dotted lines indicate conditions selected for using in subsequent assays. OD values shown are the means obtained in three tests of five positive serum samples against M41, H52, CK/CH/2014/FJ14, CK/CH/2014/JT1, or 4/91 or five negative serum samples from SPF chickens. P/N values represent the ratios of mean OD values obtained for positive sera to those obtained for negative sera. Error bars represent the standard deviation (SD).

To optimize the pELISA, two-fold serial dilutions of the synthetic peptide and different dilutions of the positive and negative chicken serum samples, which had been confirmed by IFA, were tested. As shown in Figure 1, the optimal concentration of peptide was found to be 0.63 μg/ml, and the dilution of serum samples was 1:200 (Figure 1), based on the criteria that the P/N value of the OD_650_ ratio between positive and negative sera was highest. At the same time, the best results could reach at 60-min incubation for serum samples and second conjugated antibody (Figure 1).

### Cutoff Value, Specificity, and Reproducibility of the pELISA

**Figure 2:**
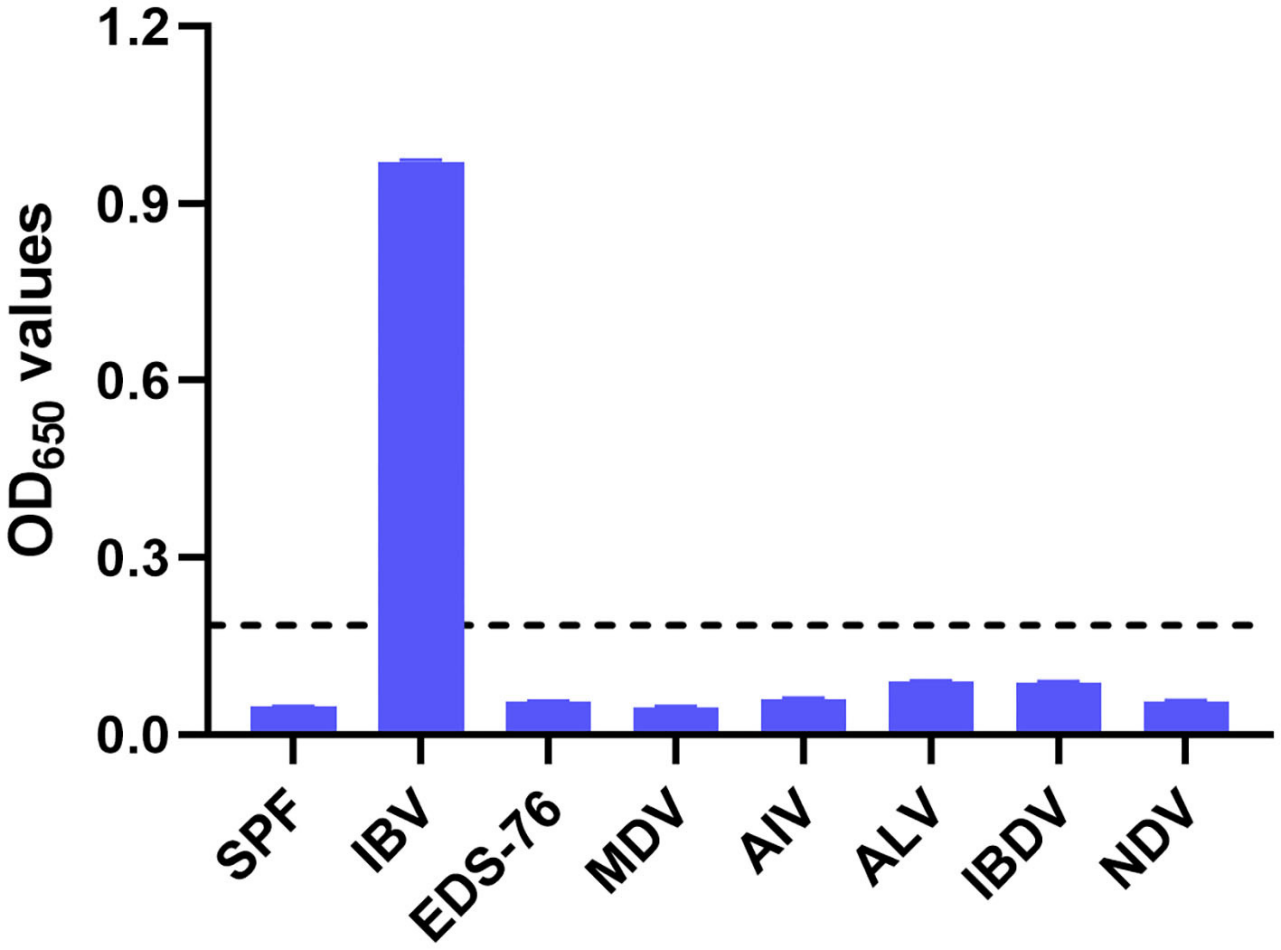
Specificity of the pELISA. Specificity of the pELISA in detecting antibodies against common chicken viruses. Antisera raised against Newcastle disease virus (NDV), avian influenza virus (AIV, H9N2), avian leukosis virus (ALV), Marek's disease virus (MDV), egg drop syndrome virus (EDS-76V), and infectious bursal disease virus (IBDV) were tested by pELISA. Their OD_650_ values were lower than the cutoff value. The black horizontal dotted line indicates the cutoff.

The 100 negative serum samples collected from SPF chickens were tested to set up a cutoff value for the pELISA. When tested at a 1:200 dilution, these samples gave a mean OD_650_ value of 0.067, with an SD of 0.017; thus, the cutoff value was defined as 0.185 (2 mean ± 3 SD). In the subsequent assays, serum samples giving OD_650_ values ≥0.185 were designated positive for IBV antibodies, whereas those generating OD_650_ values <0.185 were designated negative. The specificity of the pELISA was evaluated by testing the reactivity of sera raised against NDV, ALV, IBDV, AIV, MDV, and EDS-76V. As shown in Figure 2, no cross-reaction between the IBV S2 peptide antigen and these sera was detected, demonstrating the specificity of the pELISA.

Next, we examined the reproducibility of the assay. Eight selected serum samples, four positive and four negative, were tested by the pELISA in quadruplicate. The interassay CV was 1.2 to 6.5%, and the intra-assay CV ranged from 1.6 to 4.4% (Table 1), indicating the high reproducibility of our assay.

**Table 1:** Reproducibility of the pELISA in detecting IBV-specific antibodies.

**Samples**	**Intra-assay Variability**		**Interassay Variability**	
	**Mean ± SD**	**CV (%)**	**Mean ± SD**	**CV (%)**
1	0.043 ± 0.001	2.3%	0.048 ± 0.001	2.7%
2	0.046 ± 0.001	1.6%	0.046 ± 0.001	1.9%
3	0.049 ± 0.001	2.3%	0.051 ± 0.001	1.7%
4	0.046 ± 0.001	1.6%	0.045 ± 0.001	2.5%
5	0.842 ± 0.016	1.9%	0.817 ± 0.038	4.7%
6	0.884 ± 0.009	1.8%	0.433 ± 0.025	6.5%
7	0.421 ± 0.006	2.4%	0.249 ± 0.003	1.2%
8	0.491 ± 0.008	4.4%	0.198 ± 0.002	1.3%

*Every sample repeated three times, SD, standard deviation; CV, coefficient of variation*.

### pELISA Detects Antibodies Against Different Genotypes of IBV

**Figure 3:**
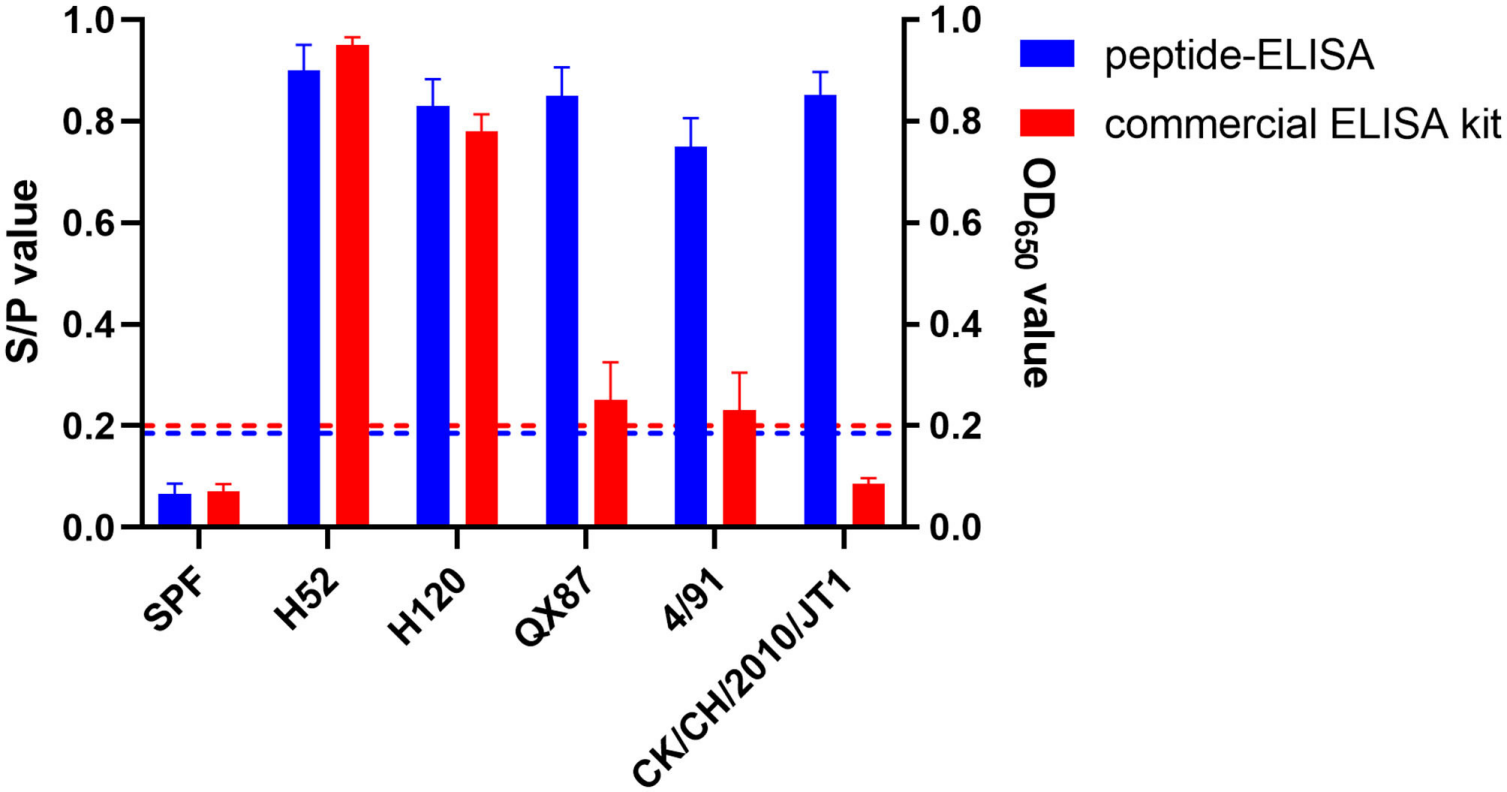
Reactivity of pELISA and positive sera against different genotype IBVs. Two colors showed the results of pELISA and commercial ELISA kits. The blue horizontal dotted line indicates the cutoff of pELISA, and the red horizontal dotted line indicates the S/*P*-value of the commercial ELISA kit. pELISA had very good reaction with sera to different genotype IBV strains, but the commercial ELISA kit gave a strong reaction with only mass-type positive sera. H52 and H120 vaccine strains belong to mass type; 4/91 vaccine strain belongs to 4/91 type; QX87 vaccine strain belongs to QX-type; CK/CH/2010/JT1 virulent strain belongs to new cluster type.

To evaluate whether our pELISA is suitable for detecting antibodies against various genotypes of IBV, chicken sera raised against IBVs 4/91, H52, H120, QX87 (QX type), and CK/CH/2010/JT1 (New cluster type) were tested in the assay. The pELISA could detect antibodies against all of these different IBVs. Interestingly, the commercial IDEXX ELISA kit could detect sera raised against the H52 and H120 strains, but showed very weak reaction with the sera to 4/91, QX87, or CK/CH/2010/JT1 (Figure 3), which indicated that the pELISA is a better option for the detection of antibodies to various IBV genotypes.

To further evaluate the sensitivity, specificity, and accuracy of pELISA, 250 serum samples from chickens immunized with H120 and H52 vaccines were tested with the pELISA, IFA, and the commercial ELISA kit. As shown in Table 2, 232 and 18 of these serum samples tested positive and negative by pELISA, respectively, whereas 233 and 17 tested positive and negative by IFA, respectively. Compared to the results of IFA, the sensitivity, specificity, and the accuracy of the pELISA were 99.14, 94.12, and 98.80%, respectively. With the commercial kit, 230 and 20 of these 250 serum samples were positive and negative, respectively. Compared to the results of IFA, the sensitivity, specificity, and the accuracy of the commercial kit were 96.57, 70.59, and 94.80%, respectively.

**Table 2:** Comparison of the pELISA, IFA, and the commercial IBV ELISA kit for 250 field serum samples.

**Serum**			
**samples**	**IFA**	**pELISA**	**IDEXX**
223 serum samples^a^	+	+	+
8,283	+	+	−
8,284	+	+	−
6,817	+	+	−
6,823	+	+	−
6,878	+	+	−
6,872	+	+	−
6,819	+	+	−
8,354	+	+	−
86	−	+	−
23	+	−	+
8,358	+	−	+
8,342	−	−	+
8,348	−	−	+
8,370	−	−	+
8,366	−	−	+
8,371	−	−	+
11 serum samples^b^	−	−	−

*+, Positive; −, negative*.

a *Number of serum samples tested positive by all three methods*.

b *The number of the negative serum samples by three methods*.

### pELISA Effectively Detects Antibody Response in Chickens

Finally, we evaluated the suitability of the pELISA in measuring the immune response elicited by the IBV vaccine. Chickens were vaccinated with IBV H52 and 4/91 vaccines, and sera were collected at different time points postvaccination and tested by pELISA. In sera from H52-vaccinated chickens, the pELISA was able to detect antibody response as early as on day 7 postvaccination, with three of the six chickens being positive, and all chickens were positive by 14, 21, and 28 days postvaccination, whereas the commercial ELISA kit could detect positive antibodies on day 14 postvaccination. In sera from 4/91 vaccinated chickens, the pELISA was able to detect antibody response as early as on day 7 postvaccination, with two of the six chickens being positive, and all chickens were positive by 14, 21, and 28 days postvaccination. By contrast, the earliest time point when the commercial ELISA kit could detect a positive antibody response was on day 14 postvaccination, whereas no positive antibody response was detected in any of the birds on day 7 postinfection. Further, on days 14, 21, and 28, only two four and five of the chickens tested positive by the IDEXX kit, respectively (Table 3).

**Table 3:** Comparison of pELISA and a commercial ELISA kit in detecting IBV antibody response in chickens^a^.

		**Day 3**	**Day 7**	**Day 14**	**Day 21**	**Day 28**
H52	pELISA	0/6^b^	3/6	6/6	6/6	6/6
	Commercial ELISA kit	0/6	0/6	6/6	6 /6	6/6
4/91	pELISA	0/6	2/6	6/6	6/6	6/6
	Commercial ELISA kit	0/6	0/6	2/6	4/6	5/6

a *Chickens were vaccinated with IBV H52 and 4/91, and sera were collected on indicated days and tested by pELISA and the IDEXX ELISA kit*.

b *The number are positive number/total number*.

## Discussion

ELISA has been used in IBV serological tests for its feasibility, sensitivity, rapidity and being suitable for large-scale use (17). In the previously reported IBV antibody detection assays, whole IBV virions and recombinant proteins were used as antigens (10–13). Although these assays have their advantages, preparation of the antigens was time-consuming and expensive. In this study, we developed a pELISA for IBV antibody detection, using a peptide based on the S2 sequence. In contrast to S1, which is highly variable among different IBV strains, S2 is highly conserved and carries conserved epitopes (7, 14, 18). Our results demonstrated that the pELISA could detect antibodies against different genotypes of IBV. Furthermore, the assay is highly sensitive, specific, and accurate compared with the results of IFA and the commercial ELISA kit, highlighting its value as a reliable assay for the detection of IBV antibodies.

For antibody detection, different methods could give different results. The same method with different antigens also gives different results (10, 19). In our pELISA, we could detect antibody against IBV on as early as 7 day postinfection. However, the commercial ELISA kit could not detect the antibody until 14 days postvaccination, and the positive value was not very strong. Our results are similar to those of Kutle et al. (20), who found that the titer of antibody to IBV was very low on 20 days postvaccination when they used the commercial ELISA kit. The reasons could be mainly because of the surface antigen variation of the virions and different coated protein. In addition, high concentration of epitope of S2 protein could have good reaction with the antibody to the conserved epitope in different genotype. It indicates that the epitope in S2 could be an important antigen in IBV immune response.

In summary, we have developed a pELISA with a synthetic S2 peptide, which could detect antibodies against different genotypes of IBV. This assay possesses sufficient sensitivity, specificity, and accuracy and has the potential to serve as a rapid and reliable method for IBV antibody detection.

## Data Availability Statement

The original contributions generated for the study are publicly available. This data can be found at: https://www.ncbi.nlm.nih.gov/nuccore/KU361187.1/.

## Ethics Statement

The animal study was reviewed and approved by Animal Care and ethics Committee at Yangzhou University.

## Author Contributions

This manuscript was written by LY and AQ. Experiment and data analysis were performed by LY, QW, ZL, and YL. Study designed by ZW, AQ, JY, KQ, and HS. All authors contributed to the article and approved the submitted version.

## Conflict of Interest

LY and YL were employed by the company JiangsuLihua Animal Husbandry Co., Ltd. The remaining authors declare that the research was conducted in the absence of any commercial or financial relationships that could be construed as a potential conflict of interest.
